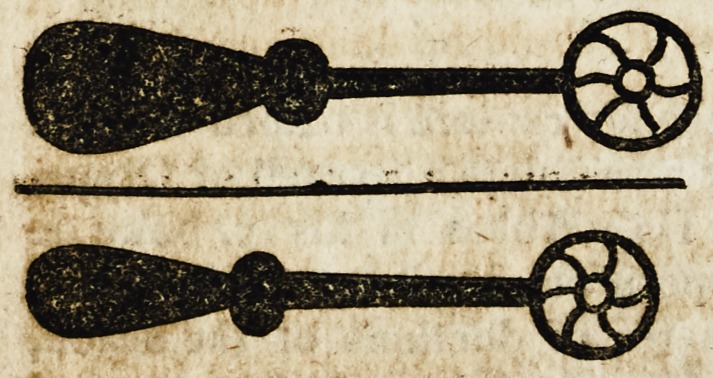# Collectanea Medica, Consisting of Anecdotes, Facts, Extracts, Illustrations, Queries, Suggestions, &c.

**Published:** 1816-05

**Authors:** 


### COLLECTANEA MEDICA,

CONSISTING OF

### ANECDOTES, FACTS, EXTRACTS, ILLUSTRATIONS, QUERIES, SUGGESTIONS, &c.

RELATING TO THE

*History or the Art of Medicine, and the Auxiliary Sciences.*

Quicquid agunt medici,
Nostri farrago libelli.

### *Further Extracts from the Black-Letter Book, published Anno* 1541, *as mentioned in our last.*

¶ Here begynneth the fourth treaty of this present questyonary, in conteynynge iiij. partycles. In the fyrste partycle is moued and solued certain questyons and dyffycultees vpon the maner of bledynge.

¶ Demaunde.

WHAT is bledyng or blode lettynge? Answere. Dyuers Auctours haue gyuen dyuers diffynycions of bledynge. Arnolde of the newe towne in his boke of partyculer operacyon that bledynge is incysyon of veynes, by the whiche incysyon the blode euacueth and the humours that rēne in the veynes with y^e^ blode. And Auycen in his fyrste fen, of the fyrste boke of his canon sayth, that bledynge is an vnyuersall euacuacyon of emptyeng the multitude of humours. And in the thyrde boke of the sayde canon he hath dyffyned that it connue euacuacyon of humours. And Galyen upon the syxth artycle of the affoe of Ypocras upon this canon, Quecunque flōmie, &c. sayth that it is the comyn helpe of pluresy. ¶ Demaunde. What euacuacion is moste surest & least daungerous, eyther the lettynge blode or the medycyne laxatyfe? Answere. After Galyen in his lytell boke that he made of blode lettynge, that lettyng of blode is the least daungerous, for it is restraynt whan we wyll, and nat the medycine, for after that it is ones taken it wyll do y^e^ operacyon. ¶ Demaunde. For howe many intencions be the bledynge made? Answere. For vi. The fyrste is for to purge; and of this intencyon sayeth Galyen in the thyrde of his Terapentycke that euacuacyon for the obiect regardeth all onely y^e^ replexion. The seconde intencyon that bledynge is made is for to dyuerte, and this intencion putteth Galyen in the seconde boke of blode lettynge: it is somtyme antyspatic, that is to say dyuersyue; and this declareth Galyen in y^e^ fyfth boke of his Terapentycke, as the flux of blode at the nose of the ryght nosethrylle, is restraynte by the bledynge of the ryght arme. And whan the left nosethrylle bledeth the blode lettynge of the lefte arme restrayneth it, for the diuersion of the blode that for the blode lettynge taketh another way, and tourneth tourneth in to other places than at yᵉ nose. And this lykewyse sheweth vs Ypocras in yᵉ fyfth partycle of his affor, where he sayth that yf the hynder parte of the heade dyd ake, yᵗ the souerayne remedy is to make the ryght veyne of the foreheade be opened, and nat onely for the euacuacyon that is made by the bledynge, but lykewyse for the antispase and diuersion. The thyrde intencyon wherfore bledyng is made is for to attray as Galyen declareth in the boke abouesayd of blode lettynge. Yf we wyll cause the menstrues of women to come we cause the sophynes of the feet to be opened, nyghe to y tyme that they shulde come, or els we apply to them ventoses wᵗ scaryfycacyons in the nether partyes. The fourth intencyon wherfore lettynge of blode is made is for to alter, as sayth Galyen in the fourth boke of his Terapentycke, and vpon the fyrste artycle of the affor, that blode lettynge vnto Lipothomie, that is to say vnto fayllynge of the hert, sodaynly coleth all the body, and restrayneth the feuer as yf it hadde slayne it. The fyfthe intencyon is for to preserue, and this intencyon declareth Galyen in the said boke of blode lettynge, and on the syxte of affor, vpon this affor, that to who soeuer yᵉ blode lettynge is good & conuenable where as he sayeth that many dyspose to periplemonie and spyttinge of blode, to quynsees, to epylence, and appoplexy, were preserued of the sayd inconuenyentes, by lettynge of blode at the sprynge tyme. The syxte intencyon is for to lyghten nature, as Galyen declareth in the eleuenth boke of his Terapentycke, the xv. chapytre, towarde the myddes of the said chapytre, sayeng that it is than better to cut yᵉ veyne, nat onely for the feuers synocalles, but also in all the other that ar of rotten humours, and to them that haue aege and suffysaūt strength therto. For nature dyspensed ouer all the body is lyghtned by cause yᵗ the thynge that greued it is taken away, as a great burden lessened and made lyght. The rest it dygereth that yᵗ ought to be dygered, and dyuyde that yᵗ ought to be dyuyded, and retourne to kyndly operacyons. ¶ Demaunde. What be they that may well bere the lettynge of blode? Answere. To this questyon Galyen in the boke aboue sayd of blode lettyng sayth that it is they that are robust and stronge, and that haue byg and large veynes, and that be nat to leane, to whyte, & tender. And contraryly the other may scantly suffre it for they haue but lytell blode, and theyr flesshe is largely euaporable. ¶ Demaunde. What folke suffreth nat blode lettynges? Answere. It is they that are of contrary dysposycyons to the dysposycyons aforesayd, as whytely coloured and leane folkes, or ouer fat and weake, yᵗ haue streyt veynes; and tendre folke, & specyally lytell chyldren afore xv. yeres, and olde folke after lxx. yeares, yf it be nat by great nede and with great cautele; and he that be nat wont to be letten blode, and they that haue weyke stomackes, and haue flux of the bely dyatryc, and people gullyng, fraungyng, and dronkerdes, & women with chylde, chyefly in the fyrste and last monethes, as vnto iiij. monethes, and after vij. monethes vnto the ende, and women havynge theyr floures; and Rasis in his fourth boke boke of his Almansor putteth to them that haue fasted and suffred hūger. The fleumatykes, & them that are wont to diseases of colde maladyes. And those that dwelleth in very colde regyons, or vehement hote. ¶ Demaunde. How many and what veynes are to be let blode in the body of mankynde? Answere. As Haly sayeth in the nynth sermon of the seconde parte of his boke, de regali dispositione, there be xxxiij. Of the which there be xij. amyd the armes, that is to wyte two medyans, two cephalykes, two basilykes, two affelleres, two cubytalles, and two seynalles. And in the head there be xiij. That is two behynde the eares, two in the angles of yͤ eyes, two organykes, two on the fūne of the heade, one on the foreheade, one on the hyndre parte of the heade, one on the nose, and two vnder the tongue. And there be viiij. in the fete, two on the knees, two sopheynes, two scyatykes, and two at the ancles. Howebeit Albucrosus putteth in all but xxv. That is to wyt xv. in the heade, v. in the armes, and v. in the legges.

The most remarkable things in the above extract is the implicit reliance on Galen and the Arabian writers; the author conceiving it necessary to produce their authority in every answer. The passage from Galen on bleeding to fainting, in order, at a single stroke, to subdue a fever, was, we fear, overlooked almost from the time of Sydenham to Robert Jackson.

Two chapters follow on cupping and leeching, called here *ventosing* and *boring*.

¶ And here begynneth the seconde partycule wherin is moued and assoyled certayne questyons and dyffyculteis vpon the maner of ventosynge or borynge.

¶ Demaunde.

What is ventosyng? Answere. It is the puttynge of boxes vpon any membre for to expuls the mater betwene the skynne and the flesshe. ¶ Demaunde. What are ventoses? Answere. Ventose is an instrument made in maner of a boxe with a streyt necke and a wyde bely. ¶ Demaunde. Wherof ought ventoses to be made? Answere. After Albucrasis they be made of thre thynges. Some of hornes, some of glasse, and some of brasse. ¶ Demaunde. Howe many fourmes is there for to vse ventoses, and what is theyr effectes? Answere. Some be with garsynge [cutting], and other without scaryfycacyon. Those that be done with scaryfycacyon draweth the mater out felyng, and yͤ other contraryly. ¶ Demaunde. What dyfference is betwene euacuacions done by blode lettynge, by ventosynge, and by snayles blode sowkers [leeches]? Answere. The moste dyfference is of blode lettynge, for it draweth the blode deper than the boxynge or the snayles, and the snayles deper than the ventoses, whiche proprely draweth but betwene the skynne and the flesshe. And therfore Auycen sayeth yͭ they purge more the thynne blode than the thycke, and more the vpperest than the nether. ¶ Demaunde. For howe many and for what intencyons are ventoses applicate with garsing vpon a mans body? Answere. For xii. intencyons. Some general,

1 nerall, nerall, and some ꝑtyculer. The generall is made to clense sensy-bly, and haue the place of a blode lettynge, whan blode lettynge dare nat be done for diuers thynges that letteth blode lettyng, as in a chylde of xiiij. yere olde, & in aged folke aboue lxx. yeres. And for this cause Auycen calleth vētoses curates of yᵉ veynes. The vii. intencyons wherby the sayde ventoses is applyed, is taken of the places yᵗ they be sette to. The fyrste is to purge the mater of the heade, and the partyes therof; and therfore they are applyed in the nawpe of the necke, and kepeth the place of the cephalyke bledynge. And therfore they be good for the dyseases of the eyes, to the infections of the face, and stynkynge of the mouth. The seconde intencyon is for to clense the spyrytual maters, and therfore they must be applied betwene the shuldres, & kepeth the meane for blode lettynge of the medyan, and therfore they be conferent to the dyseases of asma, palsye, & spettynge of blode. The thyrde intencion is to empty the mater that is conteyned in the nutrytyfe membres; and therfore shulde they be applyed to the raynes and to the loynes, and there they take the place of basilica, & therfore they auayle to the opylacyons, apostumes, and dolour of the lyuer of the reynes, and scabbes of all the body. The fourth intencion is that it is applied in the myddes of the arme, for the ache & paynes of the parties therof. The fyfth intencyon is for that it is applyed in the myddes of the thyghes and the legges nygh to the ancles, and applyed there is in the stede of the blode lettynge of the sophynes, and therfore they prouoke the floures to women, and causeth them to pysse, and easeth the paynes of the matryce and the bladder, and cōfereth to the gowte of the fete & euyll sores.

¶ Demaunde. For howe many, and for what intencyons the ventoses applyed without scarificacyon? Answere. But for one generall intencyon, and for xi. partyculers. The intencyon general is for to drawe, and the particulers do vary after the places that they be applyed to. The fyrste place is vpon the ypocondres to reduce & dyuert the blode of the nosethrylles after Galyen in the fyfth of his Terapentyke sayenge that whan the ryght nosethryll doth blede, for to staunch it yᵉ ventose must be applyed vpon the lyuer, and whan one bledeth at the left nosethryll it must be applyed on yᵉ mylt. The seconde place where they be applyed is vnder the brestes for to staunche and dyuerte the floures of women, as Ypocras sayth in yᵉ fyfth of his affo, and as Galyen declareth in the begynnynge. The thyrde place where they ought to be applyed is on the interyour parte of the heade for to reyse the euela, & to staunche the rewme. For to drawe the depe mater outwarde as Galyen declareth in the xiij. boke of his Terapentycke, and for that cause they be often applyed vpon yᵉ appostumes that be in the clensyng places, the which Auycen byddeth to be drawen out as moche as may be. Lykewyse they be applyed for yᵉ same cause vpon yᵉ thyghes, for to prouoke floures in women. And also nyghe to the appostumes of the ioyntes, to wᵗdrawe & deffende that the sayde appostumes do nat brede, and to put ferof the humours frō yᵉ sayde ioyntes. The fourth place to apply them is vpon the bredyng of synewes, in palsy, for to heat them, as Auycen sayeth in in the thyrde boke of his Canon in the Chaptour of palsy. And Galyen in the thyrde boke of yᵉ interyours, where as he proueth agaynst Archygenes that the brayne is pryncyple and begynnynge of the vertue anymall. The fyfth place to apply ventoses is vpon the bely in colyke passyon, for to resolue and vnuapen the vētosite, and cease the payne. The vi. place is vpon the matryce, and vpon the bowelles for to reduce and withdrawe them to theyr places, as Auycen sayth in his thyrde canon. The vij. place is vpon the rybbes & lyke bones for to reduce and retourne them in to theyr places, whan they are broken or disioynted. The viij. place is vpon the wayes and poores wherby the vryne passeth from the reynes to the bladder, as Auycen sayth in the thyrde boke of his Canon. The ix. place is vpon the eares & gappes of depe woundes, for to drawe out the fylth or other noyaunce yf there were any. The x. place is vpon the necke for to enlarge the wayes of the breth and of the meate. The xi. place to apply ventoses is vpon venymous bytings and blaynes to drawe out the thycke venym. ¶ Demaunde. Howe ought they to gouerned that must be ventosed before & after it? Answere. To the fyrste answereth Galyen in the thyrde boke of the cretyke dayes, and the same proueth Albumazer ī his great introductory that the chosen dayes for to applye ventoses is whan the moone is ful & nat in the wane. For as the moone encreaseth in lyghte, lykewyse encreaseth the humours within the body, and as it waneth so descreaseth yᵉ humours and withdrawe them inwarde. And therwith it ought to be an australl day, that is to say hote and moyste, and the ventoses ought to be plyed from two of the clocke unto thre. And after y intencyons of doctours; fyrste yᵉ place ought to be bathed and fomented (which should be ventosed) with warme water yf the blode be thycke, but yf it be thynne it is nat nede at all, for it shuld be daunger of to moche resolucyon, and that the strength shuld weyked. And it is to note, that neuer scaryfieng ought to be made but fyrst ye must put to the ventose drye, bycause the blode must be drawen or it be voyded. As to the seconde question it is to be noted as is afore spoken that there be two maners of ventoses. Some be of horne, and some of glas. They of horne are applied in suckynge. They of glas with towe put in to the ventose, and fyre in the twoe and layde on the flesshe, than the fyre quencheth where the ventose taketh. Or after Albucasis, take a lytell candell of waxe and gyue it a lytel stey belowe that it may holde ryght vpon the flesshe, and lyght it, than set on the ventose, and the candell wyll quenche and the ventose take holde. And the Cyrurgyen oughte with his handes to rubbe all aboute the place to moue the blode to it. As to the thyrde questyon after that ye haue applyed and set to the said ventose by two or thre tymes yf it be nede whan it is taken away ye ought to make certayne scaryfycacyons very depe with the rasour, and than wype and drye the blody place, and than ones agayne set to the ventose as ye dyd before, and kepe it on halfe an houre tyll it be halfe full of blode, and then take it away and wype the place, and set it on agayne, and holde it there more or lesse till ye haue suffycyently halfe a pounde of blode, or to a pounde, after the tenour of the strength the quantyte of the replexyon. And yf after the fyrste apposycyon after the scaryfycacion yf it blede nat wel rub the place w^t the mouth of the ventose, or gyue it small fyllyps with your nayle, and garse it a newe that it may blede well, and whan it hath ben ventosed wype and drye the place, and than anoynte it with oyle of Roses or other oyntement to mytygate the smert, and gouerne the pacyent as is aforesayde of them to be letten blode. ¶ Demaunde. Shal they be set vpon brestes of women or other softe place? Answere, Nay, for daunger that it do nat entre to depe in quantyte, & may nat be had agayne. ¶ Demaunde, Yf the ventoses wyl nat hold whan they be set on, what ought y^e Cyrurgyen do to make them faste? Answere. He must bath and foment the place all about with warme water in such wyse & so longe that the eyre entre nat. ¶ Demaunde. Is it nedefull for to contynue & kepe them longer? Answere. No, specyally aboute the pryncipal membres that are the mynes of strength; for behynde the necke they hurte the mynde, and behynde the shuldres they anoy the herte, & in the ryght ypocondre they noye the lyuer.

¶ Thus endeth the seconde partycule of this treatyse.

### ¶ Here begynneth the thyrde partycule of this treatise, where as be moued and assoyled some dyffycultees and questyons vpon the maner to apply blode suckers or horse leches.

### ¶ Demaunde.

Wherfore are horse leaches applyed? Answere. For to vnderstande y^e solucyon of this questyon is to be noted what horse leches be. They ar wel knowen to be certayne lytell blacke wormes lyke to Myce tayles and haue small yelowe streykes on theyr backes somwhat brownysshe vnder the bely, and to the question they are put and applyed to drawe or soucke as is beforesaid. ¶ Demaunde. Whiche are the blode suckers that ought to be chosen; and whiche are holsome; and whiche are daungerous and oughte nat to be applyed in any wyse? Answere. They that be good are foūde in clere waters; and they that be of a lothsome coloure with great heades, and that be rotten, and founde in noughty waters be daungerous, euyll, and venymous. ¶ Demaund. To what bodyes and to what membres ought they to be applyed? Answere. They ought onely to be applyed in bodyes voyde of replexyon, for in cacechymyke bodyes and replete they ought neuer to be applicate as touchynge the places and membres that they oughte to be put to, they are applyed but onely to suche places as ventoses can nat be set, as to synewes, in the lyppes, gūmes, & in places drye and scarce of flesshe, as the fyngers and ioyntes. And Thederyc wylleth that somtyme they be set vpon apostumes of the clensynge places, whiche are of dyffycyle curacyon and maturacyon; & some wyll haue them set on emoroydes for to open them. ¶ Demaunde. In howe many maladyes are blode suckers good? Answere. Auycen sayeth y^t they be good to scabbes, scabbes, to emoroydes, and to apostumes of the clensynge places as it is sayd. ¶ Demaunde. How shulde blode suckers be applyed? Answere. They ought nat to be applied whan they are new taken, but kept in fresshe clere water all a day tyll they haue purged of all that was in theyr belyes. And than rub the place that ye wyll put theym to tyll it do were ruddy, and wasshe it or anoynte it with a lytell blode, or garse it with a rasonre that some blode yssue, & than put them to with a rede or your handes, and put them in two or thre places as nede shall be. And whan they haue wel sucked and drawen tyll they be full, they wyll fall of by themselfe; or els put a lytell vyneygre on theyr heades, or whyte salte, or aloes, or seperate theym with a horse heare or a fyne threde. ¶ Demaunde. Howe shall the place be ordred after that they are fallen of? Answere. Rub and wasshe it with salt and vyneygre. ¶ Demaunde. Yf after the extraction and fall of the worme there folowe emororgie or to great flux of blode, what ought the Cyrurgyen to do? Answere. To staunche it with a playster of Bolarmynyke, galles, balastye, & other that stau͞che blode. ¶ Demaunde. How ought he to be ruled that hath ben blode sucked after that they are fallen of? Answere. He ought to be ordred as they that be let blode, as it is wrytten in y^e^ fyrste partycle of this treatise; and he ought to take tryacle for doubte of ventosytees that blode suckers do brede.

¶ Thus endeth the thyrde partycle of this present treatyse.

The next chapter, on Cauterizing, is curious, not only as exhibiting the instruments of those days, but it shows that three centuries ago the French preferred the actual to the potential cautery.

### ¶ Here foloweth the fourthe partycle, where as be moued and soyled other dyffyculteeς touchynge the maner of cauterisynge or fearynge.

### ¶ Demaunde.

What is cauterysacyon? Answere. It is an operacyon made w^t^ fyre artyfycyally in y^e^ body of man for certayne vtylytees. ¶ Demaunde. Howe many maners of cauteres be there? Answere. Two maners. Some are actualles and they appere sodeynly in effecte, as they that are made w^t^ instrumentes of metall, and brennynge; or with the rote of Arystologie, or of Affodylles that are sore hette, or with water, or with sethyng oyle layde to the place, co͞nyngly & nat at aduenture. Other are potencyall whose operacions are nat so sensible nor so sodayne, but appereth afterward as they that be made with bre͞ning or ruptycke medecines. And there is two maners of theym. Some are of stronge oppressyon, and maketh scarres as lyme & sope and anacardus. Some other thyrleth more lyghtly and make no scarres, but blysters as canterides, fla͞mule, and pantalupina. ¶ Demaunde. Which cauteres are the surest, the actualles, or the potencyalles? Answere. The actuelles, because y^e^ action of fyre is moste simple. And also it hurteth lesse the nexte partyes, and pryncypall membres than the action of rupture; for it is greatly suspecte to the pryncipall membres, and therfore it ought nat to be applyed, but yf case be that the pacyente were faynte herted and durst nat abyde the fyre; and in case that ye wolde apply cauteres lastly and for to purge; for in suche case the rupture for the payne that it maketh and for the byg scar that it leueth, and in weykynge of y^e^ place is cause of bygger flux of blode. ¶ Demaunde. Which is moste profytable to make actual cauteres with golde or with yren? Answere. In pryncypall and tendre membres, as the eyes, it is better to do it with golde than with yren. Howbeit in other mēbres it is more behouefull to do it with yron as sayth Albu. For the fyre may be better estemed in y^e^ yren than in the golde or in syluer bicause of theyr colours, but yf it were a goldsmyth that is wonte therto. ¶ Demaunde. Yf actuall cauteres be necessarye, and to whome, and wherwith? Answere, Fyrste they be necessary to conserue helth and to heale diseases, and kepeth the rowme of profytable purgynges, as blode lettynges, and clensynge by medycyns laxatyues, in such that may nat suffre them. And the rest that remayneth after the purgynges it correcteth in great and stronge dyseases, where as it is wonte to be gyuen. Secondly they be necessary and conuenable to be gyuen in all dysposycyons of maladyes; and specyall in materyall maladyes, saufe in suche as are hote and drye, wherin they do many euylles; and that it is true that they be profytable in the other fyrste dysposycyons, and colde dysposycyons and moyste, in as moche as it contraryeth them holly. Thyrdly in hote and moyste dysposycyons in which suppose that holly they do nat contrary, neuerthelesse they contrary accydentally; in as moche as it voydeth the cause of the maladye. Howebeit it is to be noted that suppose a cautere be a profitable remedy & very conuenable, yet it is nat nowe adayes so moche in vse as it was wont to be for the abusers of the arte and that exercysed it, the whyche indyfferently and in all dysposycions, that is to wyt in replection or otherwyse apply them. And it is euyll done, & many euylles foloweth therby. And therfore good Cyrurgyen beware ryght well, that in a persone ful of humours good or bad neuer to apply Cautere without precedent purgacion. ¶ Demaunde. For howe many and what vtylytees are cauteres made and ordeyned? Answere. For vj. vtylytees. The fyrste to comforte the membres, for they chaufe and drye the membres that were dulled with colde and humydyte. And therfore Galyen sayeth of the auctoryte of Ypocras, that the drye thynge is nerest the hole thynge, and the moyste thynge ferdest of. The seconde vtylite is to withstande & deffende the membre from corruption. And therfore Auycen in his fourth boke cōmaundeth them to be done rounde aboute the estionoenes sores spredyng or compassynge, and to corrupte bones. The thyrde vtylyte that Auycen putteth is to resolue the coarted maters in any membre; and therfore byddeth Albucrasis and Haly Abbas that they be applied to the paynes of the ioyntes, & great doloure of y^e^ heade. The fourthe vtylyte is to staunche the blode, as Auycen putteth, and Galyen in the fyfth of Terapentye, bycause

 they they make scarre. The fyfth vtylyte is purging olde fluxes as the eyes, & of all the body, & this vtilite putteth Arnolde of villenensis. And for that cause be the cetons & canteres done (behynde the necke,) and in the fontenelles of the lacertes where as one is deuyded from the other) vnder the sayd lacertes a two or thre fyngers fro the ioyntes. The syxth vtylyte that Galyen putteth is to entre-breake, and intercyde the matter. And for that cause are the veynes of the temples canterysed, bycause that the mater rẽne nat in to the eyen, and in ruptures that the bowelles shall nat descende, and in the cyrcuyt and next places to wycked sores. And of this vtylyte Arnolde of vylle. maketh an afforysme, where he sayeth y<sup>t</sup> the rennynge can nat be diuerted nor yssue kyndly, and that his abiden may be cõpetently clensed by canteres. The vij. vtylite is for to drawe out the superfluytees. This vtylite the cõmon vsage approueth by operacyon of apostumes by canteres, and by cuttynge of kyrnelles, & extyrpacyon of flesshe quycke or deade. ¶ Demaunde. Which are the places and partycle of actual canteres? Answere. After men of this tyme there be viij. The fyrste is ap-plyed to the toppe of the heade wherto the mayster fynger may reche begynnynge a spanne frõ nyghe to the rote of y<sup>e</sup> nose stretch-ing vpwarde; & the doctours wyll that there ought to be applied a rounde cantere with an oliuare for to resolue the brayne and dyuert the rewpose maters in the subiecte places by lowe, and some depe them to the bone, & other rase & make bare the fyrste table of the scul. Howebeit Alb. approueth it nat, and the sayde can-teres applyed to the sayd places auayleth to ydlenes, fallynge euyll, paynes of the heade, and to rennynge of the eyes, to ptysyke, and to all rewmes.

¶ Demaunde. Whiche and howe many be there of actuall can-teres, wherto they be vsed, and what shapes haue they? Answere. Dyuers auctores haue vsed and dyscryued the forme or shape of certayne. Wyllyam of Salicet discriueth vi. or viij. Lanfranc x. Henry of Mundeuyll vij. Howbeit of all cõmon canteres Guydon dyscryueth but vj. whose names & formes foloweth. ¶ The fyrste is called Cultelere (of Constean) that is a knyfe, and it is of two maners; one is called Dorsall bycause it hath a backe & cutteth but on the one syde; and the other is Ansall bycause it is made in maner of a swerde cuttynge on both sydes. And with this Cultelere be the superfluous flesshes cut, and apostumes are opened, and the sores vlceres rectified. Of the which Culteres the shapes or fourmes are suche as be here fygured.

¶ The seconde cantere is named Oliuare bicause it resembleth a kyrnell of Olyue, as sayeth Haly Abbas in his ix. boke de regali dispositione in the seconde party, and chapyter of doctions of the heade, and nat lyke to Olyue leafe as wened Lanfrancke, Salicet, and Henry. Also his operacyon declareth it, the shape is thus.

¶ The

¶ The thyrde Cantere is called Dacteler bycause it is in semblaũce of a Date stone; and it is bygger than is the Olyuare; and the fourme is suche.

¶ The fourth is named punctuall, which hath the poynte sclendre and rounde; & it is of two maners. One is made with a rest or platte, bycause it shall nat perce thorowe the skynne; and with this there be Canteres to the dyseases called knottes in the fontenelles, and in the armes and legges. The other is playne & longe in maner of a beme of the sõne; which is applyed with a quylle; The fourmes of them are suche.

¶ The fyfthe is called a cantere subtile wherwith the Cetons are applyed with small tonges brode and perced. And this is lyghter and more durable.

¶ The quyll.

¶ The plate.

¶ The fourme of the seconde is suche; that the punctualles bycause the name of punctualles do fall often, & haue nede of byndynge more tydeous than these be; the fourme is suche.

¶ The vj. is called Cyrculare, whiche hath v. adiutours to make v. canteres ad nodulum with plate perced of v. holes af- this fourme followynge.

¶ The tonges.

¶ Demaunde. Howe and in what maner ought the canteres be ap- plyed? Answere. They ought to be applyed in the fourme as folow- eth. That is, fyrste the place must be sought where yᵗ they shall be applyed, and wype it wel and drie it, than blysse it, after take your platyne or quyl and apply them all colde; but ye must nat let them lye long; and than gyue the canteres to the workeman that shall applye them all hote and very flamynge flamynge, so y[t] the pacyent se them nat. And let them be applyed vpon the sayde places in reuoluynge them contynually from one place to another that they cleue nat to the flesshe, tyl the rednes begone. And they must be harder pressed vpon the bones than on the synewes, and more lyghtly; and let it be done as oft as nede shall be. ¶ Demaunde. In what tyme, and in what hour ought the canteres to be applyed? Answere. After Galyen in y[e] thyrde and thyrtene boke of his Terapentyke, at all tymes and at all houres as necessyte requyreth; so that the body be clene and nat full of humours. ¶ Demaunde. Howe longe ought they to be kepte open after the canterysyng? Answere. After y[e] doctryne of Rog. & of those maysters by y[e] space of xl. dayes or more, by the space of thre monethes; for that is the laste terme of apostumes as Ypocras sayth in the vj. partycle of his afforysmes, and seconde of the pronostyces. And the cause is, for y[e] vertue confortatyfe entreth by the canteres by the foresayde tyme is euaporate, and the place weyked; and also there abydeth replexyon of euyll humours by the sayde openynge. ¶ Demaunde. Howe ought the place to be kept open after the canterysynge? Answere. They must be kepte open w[t] tentes or knottes of waxe, or with water in the whiche is steped and dyssolued the vertue of Euforbre, or scamony, or colloquintida, or of elebora, after the kynde of the humour that shall be purged, or with a pease, or a nut made of the wood of yuy, or of Gentian; and ouer it lay a cole leafe, or an yuy leafe; & ouer that a lynnen cloth thre dowble, and a platyne of brasse or laton, or of syluer bounde theron, and be remoued twyse or thryse a day. ¶ Demaunde. Howe must they be ordred that shal be canterysed? Answere. Thus, Fyrste or they be canterysed they must be conforted, and to them declare the vtylytees and goodnes that canteres wyll do to them; and yf it be nede to make to holde hym fast, and to bynde hym well. And after that they be cantercised ye must apply on the sayde places oyle of Roses (w[t] whyte of an egge well beaten togyder and well incorporate) by the space of foure dayes. And than apply vpon it a maturatife made with butter wel wasshed and vnsalted; and a lytell wheate floure, or with some other vnctuous thynge, and swete without salte vnto the scar be fallen; and than to be dressed & healed as vlceres be; saufe onely that yf ye wyl kepe them open for to purge y[e] humours and the vaporous fumes, or that y[e] place had ben opened longe aforehande. For whiche thynge it shulde nat be sure to close it without that it were euacuate by another place, for it shuld be daunger that the humours that were wonte to rēne in the sayde membre shulde remayne within; and that peraduenture it wold deryuate to other membres and do more harme than was before. ¶ Demaunde. Is it of necessyte that after it is closed to open it agayne, yf it be lefull to open it in y[e] same place? Answere. Yes, or in another membre nere to it, or to the next place, as Arnolde of newe towne sayth in his parabolles. ¶ Demaunde. Yf they that be canterised with potenciall canteres may be ordred as they be canterysed w[t] actualles? Answere. Yes saufe that they shal nat be bounde. And also those that blyster make no scarre, whiche must be well applyed, correct, and and reprymate of theyr malyces. And after that the blysters be reysed perce theym with cysours or a nedle, and lay a coleworte leafe theron; and couer it with lynnen, and ordre it as ye lyst. And bycause that they be nat blystred nor make no scar thus they fall within vij. dayes.

¶ Thus endeth this present questyonary made in the honour of Almyghty God; & profyte of yonge studyents in Cyrurgery, wyllyng to apply their study in y^e same arte.

The above is a most valuable specimen of ancient surgery, and of the manner in which it was taught. The catechistic form, and the authorities by which every answer is supported, almost like scripture proofs, seem to imply that the clergy of those days had the control of surgery as well as of every branch of science. The book is a small quarto: the only paging is an alphabetical letter to every fourth page, inserted at the bottom, and numbered as the alphabet is repeated.

---

Having, in a former Number, given, from the Medico-Chirurgical Transactions, a case of the true Elephantiasis of Aretæus, we shall, in the present, transcribe, from the same collection, a case of the Barbadoes Leg, or, as it is called in the island, the Rose. Our readers will, by these means, be familiar with the wide distinction between two diseases which have nothing in common but their name.

"The disease which has long been familiar in Barbadoes until within a very few years, and almost exclusively confined to that island, and which has been perhaps with impropriety denominated elephantiasis, has of late spread with unexampled rapidity through the whole of the West Indies. It is more particularly prevalent amongst the Blacks, although a considerable number of the white population are sufferers from its attacks. The parts most commonly affected are the inferior extremities; but the arms, penis, scrotum, and even the viscera, are not unfrequently the seat of the disease. The symptoms by which the elephantiasis is characterized, both in its acute and chronic form, have been detailed with such great precision by various writers, that it supersedes the necessity of any further description in this place; my object being merely to relate a singular instance of this disease, as occurring in the scrotum, which lately came under my observation. The enormous magnitude this part acquired, is, I believe, unprecedented in the records of medicine. It is not, however, merely on account of its singularity, that I have been induced to draw up the following history; but because I consider it a case of considerable practical importance, for, however rare such cases may hitherto have been, they are at present by no means unfrequent, and their number seems to be increasing almost daily.

"Montserrat, a negro, aged about 30, belonging to the estate of the late John Bourryau, Esq. received, when a boy, a kick on the right testicle from a mule. The testicle swelled, but, by the application of medicines, it was soon reduced to its natural size.

Some

Some time after this, he first became subject to the rose (the name by which the elephantiasis is called throughout the islands), which produced a permanent enlargement of both his legs. Some years elapsed before the scrotum became the seat of the disease; but, from his ignorance of dates, I am only enabled to obtain the following very imperfect history of the progress of his complaint. Five years ago the scrotum is said to have been somewhat larger than his two fists. Its increase afterwards was gradual and progressive, and did not incapacitate him from working, until within the last two years and a half. Since that time it has enlarged rapidly; and he has remained in almost total confinement, being unable to remove himself and burden from his habitation.

"Although prepared by descriptions for the scene I was about to witness, yet it was not without sentiments of the most lively commiseration and astonishment that I first viewed this singular production in October last. Language is inadequate to convey a satisfactory idea of the magnitude and appearance of this tumour; and all my efforts to procure a drawing of it proved unsuccessful.

"On removing his petticoat, there was exposed to view a tumour of rather an oval form, seemingly suspended from and greatly stretching the abdominal integuments and spermatic cords, reaching backwards to the verge of the anus, and descending to within an inch or two of the ground. It measured longitudinally from the symphysis pubis to its base 29 inches, circularly 43. The spermatic cords could be distinctly felt, somewhat enlarged; the penis was completely enveloped; the urine was discharged in a full stream, and without difficulty, at an orifice situated nine inches below the pubis; on stretching this laterally, the extremity of the penis could be seen at the distance of about three inches; this canal was formed by an elongation and distention of the prepuce. The surface of the tumour was equal, smooth, with superficial veins; the superior part thinly interspersed with hair; the inferior scaly at times. The integuments felt extremely thickened, but were not of equal firmness, and retained for a time the impression of the finger. His appetite was good, his bowels regular, and his general health unimpaired. He informed me, that, when on his back in bed, and under the impression of lascivious ideas, he was subject to erections of the penis, at which times this member would project an inch or two at the orifice above mentioned; but that they were never terminated or attended by seminal emissions.

"After carefully examining the scrotum, I informed him, that, in my opinion, no internal remedies or external local applications could possibly lessen or alleviate his disease; neither could any operation be performed short of removing the whole tumour, which would necessarily be attended with considerable hazard. He replied that life was become burdensome to him, that he would rather die than remain longer in his present situation, and that he was ready to submit to any operation how great soever the risk. My friends, Doctors William and Thomas Swanston, having done me the favour to visit him, and concurring in the opinion I had expressed, it it was resolved that the operation should be performed; but the weather being extremely unfavourable, he was recommended to wait until it should become more cool and settled. On finding that there was a possibility of his being relieved, he became so importunate to have it done immediately, that, with a view to quiet his great impatience, a large blister was applied on each side of the scrotum, and kept open by the unguent sabinæ; a seton was afterwards made in each side; but, notwithstanding these discharged freely for nearly six weeks, no diminution of bulk was observable.

"The weather being now mild, I performed the operation on Sunday the 5th of December, in the presence of Doctors Swanstons and Caines, who very obligingly favoured me with their most valuable counsel and assistance. The only difficulty that seemed to occur was how the penis could be saved: with a view to effect this, an incision, two or three inches in length, was made, commencing from a little below the symphysis pubis; the penis was by this means exposed, and, the extended præputium being divided, a flexible catheter was introduced into the bladder; all our effects to accomplish this having previously failed, owing to the retrocession of the penis. The spermatic vessels of each side were next laid bare, and secured by temporary ligatures passed round the cords. The incision was continued backwards to the verge of the anus, and the dissection carried upwards towards the penis. The tumour being removed, the spermatic arteries were secured separately, and the upper ligature slackened, but allowed to remain in case of need. The integuments were brought together by a few stitches and strips of adhesive plaster, and were sufficient to surround the root of the penis; so that this member was the only part that remained uncovered. The hæmorrhage during the operation was inconsiderable (except from a branch of the left pudic), the precaution being afterwards used of passing a ligature round the larger arteries previous to dividing them. My patient recovered without experiencing the most trifling unpleasant symptom. The wounds in the groins and in perinæo were firmly united at the end of three weeks, but the penis was not completely cicatrized before the beginning of April.

"On examining the tumour after its removal, the testicles were found to occupy their natural position: the left one was about the size of a hen's egg; the tunica vaginalis of the right side contained three pints of water, and the testicle was considerably diminished. The right side of the scrotum being opened, the integuments at the upper part were about two inches, nearer the base they increased to four inches and a half in thickness; a fluid oozed from its substance, and the cavity was filled with a gelatinous matter and fluid, which also became gelatinous on cooling. The tumour weighed 70lbs. There was nothing peculiar in its structure.

"Early in February, Montserrat commenced taking Fowler's solution, with a view of reducing the enlargement of his legs. This remedy was tried at the suggestion of Dr. William Swanston, in whose hands its use has been attended with considerable advantage.

tage. Arsenic has been long employed in the East Indies for the cure of elephantiasis (vide Asiatic Researches), and Montserrat appears to have derived benefit from it. His legs are somewhat less, and he says they feel slacker and much lighter.

"In similar operations, if the incision be made on each side of the penis, and the skin dissected inwards, perhaps a quantity sufficient to extend round the penis may be preserved. This would greatly accelerate the cure.

"Having no instances of a like operation to direct our judgment, and ignorant in what state the testicles would be found, (for the absence of emissions seemed to indicate that they had become useless,) no attempt was made to preserve them. Besides this, the thickness of that part of the integuments which must have been left as a covering, appeared to forbid the hope of their readily uniting, and the consequent inflammation and swelling of the testicles, would have added greatly to the danger of tetanus. There may be cases, however, of a more favourable nature, where it may be thought advisable to save these glands. I have said that these enormous swellings are now very frequent, and are becoming more so every day. I have a patient on the adjoining estate, and there are several others in the island, in whom the scrotum is from one-half to two-thirds as large as in the case above related; and there are numerous instances of enlargement and thickening of the scrotum, which are, no doubt, the same disease in an incipient state. When yet small, I have found setons on each side useful in reducing the swelling, but I fear such diminution will be merely temporary.

"Morgagni, in letter 43, article 42, quotes some observations from Waltheius, in which the scrotum and penis are described to be so tumid, that the latter extended itself down to the knees, and the former below them; the thickness of each of these parts corresponding to this length. On examination, the skin was found to be three times thicker than natural, and the weight of the tumour amounted to near 50lbs. Morgagni received the print of a similar case from Syracuse. There is also one of 60lbs. weight spoken of in the History of the Royal Academy of Sciences of Paris, for the year 1711. As I have not an opportunity of referring to these works, I am ignorant of the particulars of the cases, and of the mode of treatment that was adopted.

"Mr. Corse describes the case of Paunchoo, an inhabitant of the East Indies, in the Second Volume of the Medical and Chirurgical Transactions (with an engraving), in whom the scrotum reached to the ankles, and was 25 inches in length, and 38 in circumference. This, as well as those related by Morgagni, are instances of the same disease; but the present is the largest, and the only case, so far as I know, in which an operation was attempted; and I trust the success which has attended its performance, may induce medical gentlemen to propose, and patients in similar circumstances to submit to, a like operation, with the well-grounded hope of experiencing a happy termination."

We have great pleasure in offering the following testimonials in favour of the Chev. Assalini's improved Instruments in Surgery, (from the Transactions of the Society for the Encouragement of the Arts, &c. vol. xxxiii. ann. 1815).

"*The* Gold Medal *of the Society was this Session voted to the Chevalier* Paul Assalini, *Physician to Prince Eugene of Munich, for his Improvements in Surgical Instruments and Operations. The following Communications were received from him: four explanatory Engravings are annexed, and a Collection of the Instruments is preserved in the Society's Repository.*

SIR,

During twenty years of medical and surgical practice in hospitals and armies on the continent, I have made improvements in many surgical instruments, and invented others, which diminish the difficulties attending operations, and render their success less dubious. The instruments have been examined by some of the most eminent surgeons in London, and their approbation of them has encouraged me to express to the Society of Arts, &c. the desire which I have to submit them to their inspection and respectable opinion, before I return to Italy.—I have the honour to be, Sir,

Your obedient Servant,

Paul Assalini.

7, *Manchester-street, Manchester-square, London.*
*October* 25, 1815.
To C. Taylor, M.D. Sec.

CERTIFICATE.

"Chevalier Assalini, Professor of Surgery at Milan, being desirous of carrying into Italy some testimony of the degree of estimation in which his ingenuity and professional merits were held by men of science and practitioners in surgery in London, we have much pleasure in complying with his request, and declaring that we are induced, from every thing we have seen, to rank his talents very high, and to consider his contrivances for the relief of wounded men very ingenious. Joseph Banks, P.R.S.
*Soho-square, London;* Everard Home.
*Nov.* 15, 1814.

"MY DEAR SIR,

"I regret that we are so soon to lose your company, nor can I let you depart from this country without testifying my sense of the gratification and information I have derived from your society. The candour and liberality with which you have communicated important professional information, the ingenuity you have displayed in the construction of various surgical instruments, and the unremitting attention which, it is evident, you have paid throughout life to the advancement of professional knowledge, have impressed

4 me me with sentiments of respect and regard for your character, with which, be assured, I shall always remain

Your sincere friend and servant,

*Bedford-row; Nov.* 17, 1814. JOHN ABERNETHY.

*To Professor Assalini.*

"I have examined the instruments of the Chevalier Assalini with all the attention in my power. They mark a mind of superior ingenuity, and one very fertile in contrivances to lessen the difficulties of operations. Those which have struck me as deserving more than common praise are, first, the double forceps, well adapted to take up an artery separately from the accompanying nerve, or when situated so as to be deeply buried.

Secondly, his forceps for aneurism are well adapted to produce the object which he has in view. This, however, is said without meaning to decide upon the superiority of such an operation for aneurism over others.

Thirdly, his instrument for making an artificial pupil, which appears to me peculiarly adapted for the purpose.

The kind notice which was taken of the labours of others, in his work on the Artificial Pupil, manifest a liberal spirit, and a mind anxious to improve the profession of which he is an ornament.

ASTLEY COOPER.

The forceps I used in an operation yesterday, and found them to answer extremely well.

*October* 8, 1814.

"Professor Assalini, having demonstrated to us the particulars of the instruments herein expressed, we are happy in the occasion of declaring our entire approbation of them.

WILLIAM BLIZARD.

THOMAS BLIZARD.

"MY DEAR SIR,

"I feel very sensibly the honour you have done me in submitting your valuable improvements in several chirurgical instruments to my inspection. To say that much mechanical ingenuity is exhibited in the contrivance of them, is the least of their praise; for they appear to me to constitute a most useful and important addition to our stock of instruments, by which many great operations may be performed with unusual safety and facility, and the hazard and suffering subsequent to various accidents may be remedied, or greatly diminished. Your portable case of amputating instruments possesses so many evident advantages, that it ought to be regarded as an important benefit conferred on naval and military surgery.—I have the honour to be, my dear sir, with great respect and esteem, your's, most faithfully, JOHN PEARSON, F.R.S.

Surgeon of the Lock Hospital and Asylum, Consulting Surgeon of the Public Dispensary, &c. &c.

*Golden-square; Nov.* 16, 1814.

*To the Chevalier Assalini.*"

Certificates

Certificates are also affixed by Mr. B. C. Brodie, Mr. R. Keate, Mr. George Young, Mr. H. Earle, Dr. George Pearson, Dr. Robert Good, Dr. Samuel Merriman, Mr. Charles Mansfield Clarke, Mr. Robert Watt, Mr. J. Briggs, Mr. Blair, Mr. Want, and other medical and surgical gentlemen.—For a description of the Instruments, see our Journal, vol. xxxiv. pages 2 and 116.

### CRITICAL ANALYSIS

## Figures and Tables

**Figure f1:**
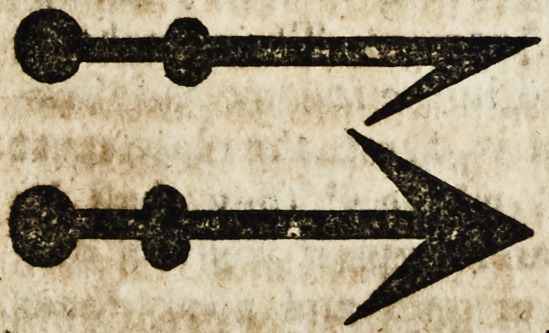


**Figure f2:**
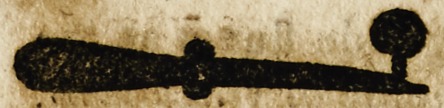


**Figure f3:**
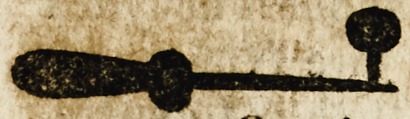


**Figure f4:**
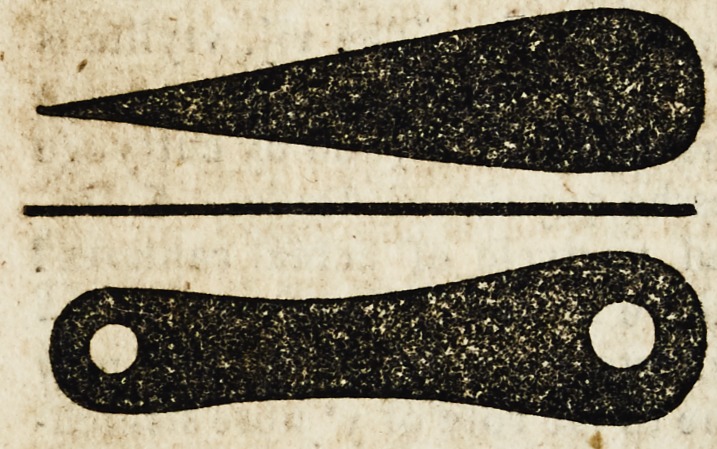


**Figure f5:**
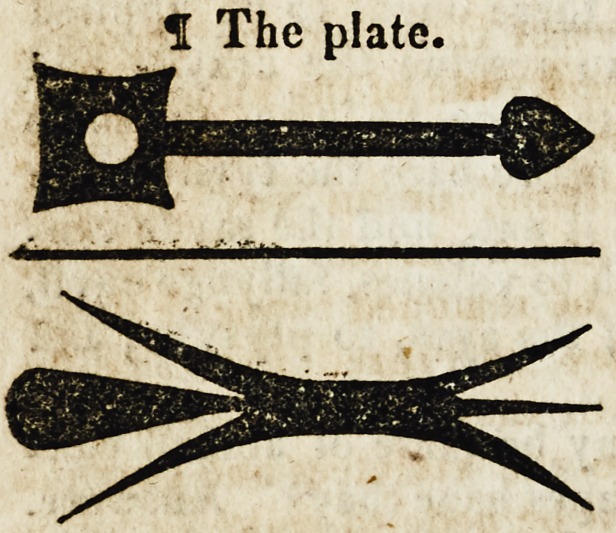


**Figure f6:**
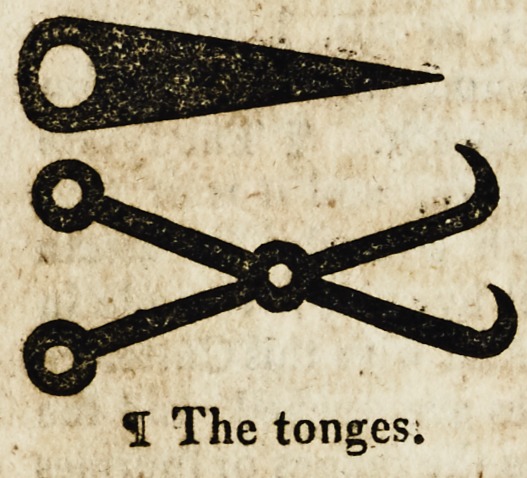


**Figure f7:**