# Errata

**Published:** 1816-06

**Authors:** 


ERRATA.

Page 400, line 11 from the bottom, for *author* read *patient.*

—— 9 ——, for *testaceæ* read *testacea.*

—— 404, —— 6 ——, for *thee vacuations* read *the evacuations.*

—— 413, —— 16 ——, for *ingenious* read *ingenuous*

